# Decreased susceptibility to cefepime/zidebactam among carbapenemase-producing *Escherichia coli* from Stockholm, Sweden with alterations in PBP2

**DOI:** 10.1093/jac/dkaf045

**Published:** 2025-02-17

**Authors:** Chaitanya Tellapragada, Chantel Dunleavy, Patrik Jonsson, Christian G Giske

**Affiliations:** Division of Clinical Microbiology, Department of Laboratory Medicine, Karolinska Institute, Stockholm, Sweden; Division of Clinical Microbiology, Department of Laboratory Medicine, Karolinska Institute, Stockholm, Sweden; Department of Clinical Microbiology, Karolinska University Hospital, Stockholm, Sweden; Division of Clinical Microbiology, Department of Laboratory Medicine, Karolinska Institute, Stockholm, Sweden; Department of Clinical Microbiology, Karolinska University Hospital, Stockholm, Sweden

## Abstract

**Objectives:**

We aimed to investigate the *in vitro* activity and genetic determinants of decreased susceptibility (DS; MIC > 4 mg/L) to cefepime/zidebactam of carbapenemase-producing *Escherichia coli*.

**Methods:**

Clinical isolates (*N* = 150) of carbapenemase-producing *E. coli* (CP-EC) belonging to seven distinct STs, isolated at a university clinical microbiology laboratory during 2019–2023 in Stockholm, Sweden were included. MICs for cefepime/zidebactam were determined using the broth microdilution method and interpreted using the tentative EUCAST clinical breakpoints (Susceptible; MIC < 4 mg/L; based on cefepime breakpoint). Whole genome sequences of the isolates were analysed with an emphasis on identifying alterations in PBPs 2 and 3.

**Results:**

Of the 150 isolates, 145 (96.6%) isolates had MICs <4 mg/L indicating susceptibility and 5 (3.3%) had MICs >4 mg/L. MICs for zidebactam alone among the five isolates with DS to cefepime/zidebactam were ≥8 mg/L. WGS analysis revealed that these five isolates were NDM-5 producers and belonged to ST405 (*n* = 1), ST410 (*n* = 2) and ST648 (*n* = 2). Presence of four-amino-acid inserts (YRIK/YRIN) in PBP3 was observed in 80/150 (53.3%) isolates, and mutations leading to alterations in PBP2 were observed in 41/150 (27.3%) isolates. Presence of other β-lactamases (CTX-M group) and/or cephalosporinases (*bla*_CMY_) did not have an impact on the susceptibility to cefepime/zidebactam. Three of the five isolates with DS had a V522I substitution in PBP2.

**Conclusions:**

Our results indicate that DS to cefepime/zidebactam among clinical isolates of *E. coli* could arise due to targeted mutations in PBP2.

## Introduction

Carbapenemase-producing *Escherichia coli* (CP-EC) strains, including those that produce MBLs, are spreading worldwide. Treatment options for the infections caused by New Delhi MBL (NDM)-producing isolates are limited. To address this challenge, pharmaceutical companies have focused on developing diazabicyclooctane (DBO) derivatives as novel β-lactamase inhibitors and/or enhancers, in combination with existing β-lactam agents such as aztreonam, cefepime and meropenem.

Zidebactam, a chemically modified bicyclo-acyl hydrazide derivative of the DBO pharmacophore, targets PBP2 and inhibits Ambler class A and C β-lactamases in Gram-negative bacilli. Zidebactam exposure results in formation of spheroplasts due to PBP2 inactivation. It was reported that zidebactam induces spheroplast formation even in the presence of β-lactamases because it is not hydrolysed owing to its non–β-lactam structure. When combined with cefepime, the bactericidal activity of the combination cefepime/zidebactam occurs through inhibition of multiple PBPs leading to pleomorphic lytic cells. Furthermore, it was also reported that cefepime binding to its target amidst β-lactamases is attributable to its faster rate of binding to target PBPs and its relative stability to β-lactamases.^1^ Cefepime/zidebactam is currently in a Phase 3 clinical trial for the treatment of complicated urinary tract infections (cUTIs) or acute pyelonephritis.

Herein, we aimed to investigate the *in vitro* activity and genetic determinants of resistance to cefepime/zidebactam among clinical isolates of CP-EC collected during a 5 year period (2019–23) at a university clinical microbiology laboratory in Stockholm, Sweden.

## Materials and methods

### Bacterial strains

Carbapenemase-producing clinical isolates (*N* = 150) of *E. coli* derived from unique patient samples during 2019 (*n* = 25), 2020 (*n* = 21), 2021 (*n* = 20), 2022 (*n* = 47) and 2023 (*n* = 37) at the clinical microbiology laboratory of Karolinska University Hospital, Stockholm, Sweden were included. Only isolates from seven STs (ST38, ST648, ST410, ST167, ST405, ST69, ST361) producing NDM and/or OXA-48 carbapenemases were included, with a minimum of 10 strains per ST. The isolates were identified through the laboratory information system and retrieved from the laboratory cryobank.

### MIC determination

MICs were determined using the ISO reference broth microdilution method, as per the EUCAST recommendations. *E. coli* ATCC 25922 strain was used for quality control, and MICs for each strain were determined in triplicate. Antibiotic powders of cefepime (Sigma-Aldrich, Sweden AB) and zidebactam (MedChem Express, Stockholm, Sweden) were used. Cefepime/zidebactam was tested in concentrations ranging from 0.06 to 32 mg/L at a fixed ratio (1:1). Isolates demonstrating MIC >4 mg/L of cefepime/zidebactam were tentatively defined as decreased susceptible (DS), based on the EUCAST breakpoint of cefepime for Enterobacterales at R > 4 mg/L (https://www.eucast.org/clinical_breakpoints). MICs of zidebactam alone were determined for those isolates demonstrating DS to cefepime/zidebactam. Concentrations of zidebactam when tested alone were from 0.125 to 64 mg/L.

### WGS and analysis

Whole genome sequences (Illumina) of the study isolates were retrieved and analysed as described previously.^2^ Briefly, quality of the raw sequencing data generated from NovaSeq 6000 (Illumina) was assessed using FastQC followed by de novo contig assembly using SPAdes (v3.15.5). ORFs from the assembled contigs were predicted using Prodigal (v2.6.3). The ORFs were searched using Diamond (v2.1.8) against the UniProt database and CARD (v3.0). Variant calling was performed using Snippy. Multiple sequence alignment was performed using MEGA for the proteins of interest. Phylotyping was performed using Ezclermont (v0.6.3), and core genome MLST of the collection was performed using ChewBBACA (v3.3.2) to call alleles in the *E. coli* scheme (v1.0) provided by SeqSphere+. The phylogenetic tree was visualized in iTOL.

All sequences have been submitted to the Sequence Read Archive (NCBI; https://www.ncbi.nlm.nih.gov); SUB15013428; PRJNA1214539.

## Results

### MICs of cefepime/zidebactam

Of the total 150 isolates tested, 145 (96.6%) had MICs <4 mg/L and 5 (3.3%) had MICs >4 mg/L, indicating DS to cefepime/zidebactam. Table 1 presents the cefepime/zidebactam MIC distribution of the study isolates. Notably, 117/150 (78%) isolates demonstrated WT MICs of ≤0.125 mg/L cefepime/zidebactam, based on the epidemiological cut-off (ECOFF) for cefepime alone.^3^

**Table 1.** Cefepime/zidebactam MIC distribution of the study isolates

ST	≤0.06	0.125	0.25	0.5	1	2	4	8	16	≥32	Total, *n* (%)
ST38	28	8	—	—	—	—	—	—	—	—	36 (24)
ST69	16	—	—	—	—	—	—	—	—	—	16 (10.6)
ST167	5	11	1	—	1	3	1	—	—	—	22 (14.6)
ST361	3	4	2	—	1	1	—	—	—	—	11 (7.3)
ST405	—	12	4	2	1	2	—	—	—	1	22 (14.6)
ST410	1	16	—	2	—	—	—	1	—	1	21 (14)
ST648	—	13	3	1	—	1	2	1	—	1	22 (14.6)
Total *n* (%)	53 (35.3)	64 (42.6)	10 (6.6)	5 (3.3)	3 (2)	7 (4.6)	3 (2)	2 (1.3)	0	3 (2)	150 (100)

### Baseline genomic characteristics of the study isolates

Figure S1 (available as Supplementary data at *JAC* Online) depicts the baseline genomic characteristics of the study isolates. Of the 150 isolates, 79 (52.67%) were NDM (76 NDM-5; 2 NDM-1 and 1 NDM-18) and 71 (47.3%) belonged to the OXA-48 group (44 OXA-244; 18 OXA-48; 7 OXA-181; and 2 OXA-484) carbapenemase producers. Four (2.6%) isolates co-produced both NDM and OXA-48-like carbapenemases. The CTX-M group of β-lactamases was identified in 88/150 (58.6%) isolates. The CMY (16 CMY-59 and 1 CMY-42) cephalosporinases were detected in 17/150 (11.3%) isolates. Nearly half of the isolates belonged to phylotype D (*n* = 74; 49.3%) followed by phylotypes A (*n* = 33; 22%), C (*n* = 21; 14%) and F (*n* = 22; 14.6%). Table 2 summarizes the key genomic characteristics of the isolates with DS to cefepime/zidebactam.

**Table 2.** Key genomic characteristics of isolates with decreased susceptibility (MIC >4 mg/L) to cefepime/zidebactam

Strain ID	Year of isolation	Carbapenemase	ESBL	AmpC	PBP3 insertions	PBP2 mutations	MLST	ZID MIC	FEP/ZID MIC
28622	2022	NDM-5	CTX-M-15	—	YRIK	V522I	405	64	32
321	2021	NDM-5	CTX-M-15	—	YRIK	V522I	410	>64	>32
420	2020	NDM-5	—	—	YRIK	V522I	648	>64	>32
39523	2023	NDM-5	—	—	—	—	648	8	8
29621	2021	NDM-5	—	CMY-59	—	—	410	16	8

FEP/ZID, cefepime/zidebactam; ZID, zidebactam.

### Alterations in PBP3 and PBP2

Alterations in PBP3 through four-amino-acid inserts (YRIK/YRIN) were observed in 80/150 (53.3%) isolates. Altered PBP3 was observed in 68/79 (86.07%) of the NDM producers. YRIK inserts (*n* = 39) were observed only among isolates belonging to three STs: ST405, ST410 and ST648, whereas YRIN inserts (*n* = 41) were observed more frequently in isolates belonging to ST167 and ST361 (Figure S1). None of the isolates (*n* = 52) belonging to ST38 and ST69 had altered PBP3 proteins. Mutations in the form of substitutions in the *mrdA* gene encoding the PBP2 protein were observed in 41/150 (27.3%) isolates. The mutations A530S (*n* = 10), V522I (*n* = 7), A543 V (*n* = 6) and K572N (*n* = 5) were most frequently observed; followed by mutations such as G601D and A543T in three isolates; A388S, L378P, V229I in two isolates; and L573Q and T173A in one isolate each (Figure S1).

### Impact of altered PBPs on cefepime/zidebactam susceptibility

DS to cefepime/zidebactam (MICs >4 mg/L) was observed in 3 out of 80 isolates (3.7%) with altered PBP3 proteins as compared with 2/70 isolates (2.8%) without PBP3 alterations. On the other hand, 3/41 isolates (7.31%) with altered PBP2 proteins had cefepime/zidebactam MICs >4 mg/L, in contrast to 2 out of 109 isolates (1.8%) without PBP2 alterations. Notably, all three isolates (28622, 321 and 420) with altered PBP2 proteins and DS to cefepime/zidebactam carried the V522I mutation. To investigate further, we identified seven isolates with the V522I mutation and determined their MICs for zidebactam alone. Table S1 summarizes the key genomic characteristics of these seven isolates with V522I mutations and their MICs of cefepime/zidebactam and zidebactam alone.

## Discussion

In this study, we evaluated the *in vitro* activity of cefepime/zidebactam against a clinical strain collection of CP-EC isolates belonging to seven frequently encountered STs, namely ST38, ST69, ST405, ST410, ST167, ST361 and ST648. Notably, strains belonging to these STs are prevalent worldwide. The contemporary strain collection comprised nearly equal numbers of NDM- and OXA-48-group–producing strains of *E. coli*, and a noteworthy proportion of these strains also carried other β-lactamases such as CTX-M-15 and CMY-59 (Figure S1). Notably, 96.6% of the isolates had cefepime/zidebactam MICs <4 mg/L, consistent with previous studies from different regions reporting over 99% susceptibility among CP-EC isolates.^4,5^

Zidebactam primarily targets PBP2 with no direct activity against MBLs and/or OXAs. It has been proposed that non-targeted mutational resistance due to stringent responses could diminish the PBP2 inhibition when zidebactam is used alone, and to mitigate this problem it has been developed in combination with cefepime.^6^ Currently there are no ECOFF values and/or clinical breakpoints established by the EUCAST for zidebactam alone. However, the EUCAST breakpoint for cefepime resistance is >4 mg/L. In this study, DS was defined as MIC >4 mg/L for cefepime/zidebactam. Given that PBP2 and PBP3 are key targets for zidebactam and cefepime, respectively, we investigated alterations in these proteins between isolates with MICs ≤4 mg/L and >4 mg/L.

Altered PBP3 and PBP2 were identified in 53% and 27.3% of the present study isolates, respectively. Five isolates (3.3%) showed DS to cefepime/zidebactam, with MICs ≥8 mg/L for zidebactam (Table 2). Notably, two of these five isolates would also be classified as resistant to cefepime/zidebactam under the tentative CLSI breakpoint of ≥64 mg/L.^7^ The V522I substitution in PBP2 along with a YRIK insert in PBP3 was a common feature observed among three of the five isolates with DS to cefepime/zidebactam. However, two isolates with WT PBP2 and PBP3 showed DS to cefepime/zidebactam, whereas one isolate with a PBP2 V522I mutation and PBP3 YRIK insert had a zidebactam MIC of 0.25 mg/L (Table S1). These findings suggest that decreased susceptibility to zidebactam is multifactorial. Notably, YRIK/YRIN inserts in PBP3 alone appear to have no impact on cefepime/zidebactam susceptibility, consistent with prior studies from India reporting full susceptibility despite altered PBP3.^4^

Single amino acid substitutions in PBP2 were observed in 41/150 (27.3%) isolates. Notably, mutations such as G601D, A543T, L573Q and K572N previously linked to carbapenem resistance, were observed.^8,9^ All isolates in this study were carbapenemase producers, but the impact of these substitutions on carbapenem MICs was not further investigated. Previously, it was experimentally demonstrated that introducing the V522I and L573Q substitutions in PBP2 of a laboratory *E. coli* strain resulted in decreased susceptibility to DBOs.^10^ The L573Q substitution was found in only one (18619) of our study isolates and it demonstrated a WT MIC (0.125 mg/L) of cefepime/zidebactam. The V522I mutation was identified in seven isolates (six producing NDM-5 and one producing OXA-244). Zidebactam MICs for the six NDM-5 producers were ≥2 mg/L, whereas the MIC for the OXA-244 producer was 0.25 mg/L (Table S1). Notably, the zidebactam MICs for isolates with the V522I mutation in this study were 2–4 times higher than previously reported values.^11^

In conclusion, cefepime/zidebactam exhibited strong activity against a contemporary collection of carbapenemase-producing *E. coli* strains. However, our findings suggest that specific mutations in PBP2 may contribute to decreased susceptibility in some clinical isolates. Further research is needed to explore the potential fitness costs associated with these mutations and to identify other mechanisms that may lead to decreased susceptibility.

## Supplementary Material

dkaf045_Supplementary_Data

## Supplementary data

Figure S1 and Table S1 are available as Supplementary data at *JAC* Online.
