# Supplementary material for: Decreased susceptibility to cefepime/zidebactam among carbapenemase-producing Escherichia coli from Stockholm, Sweden with alterations in PBP2
Source: J Antimicrob Chemother. 2025 Feb 17;80(4):1137–40. doi: 10.1093/jac/dkaf045 (PMC11962383; doi:10.1093/jac/dkaf045)
Supplement: dkaf045_Supplementary_Data [file dkaf045_supplementary_data.zip › FigureS1_ JAC.docx]

PBP3 Inserts

PBP2

MIC Values


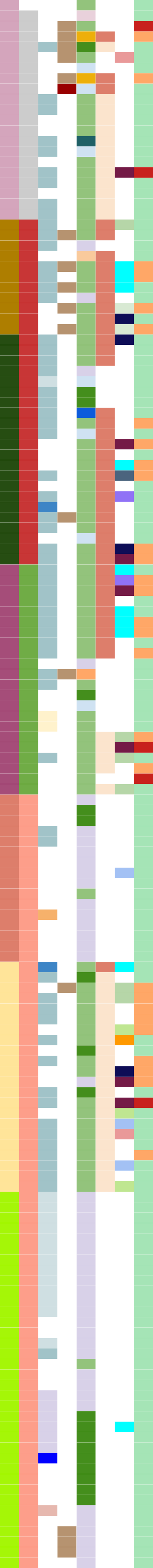


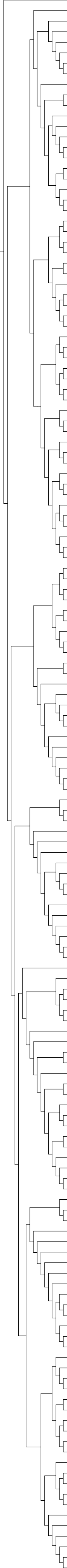
1022

STs

Phylotype

CTX-M

pAMP-C

Carbapenemase

9622

29621

14920

15523

Figure S1: Key genomic characteristics of the study isolates.

16923

43922

14820

11923

21722

16120

34422

50522

36323

28923

5422

321

2520

9422

25321

9721

38623

15623

40023


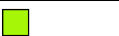

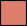

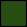

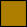

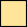

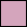

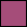


**MLST Types**

38

69

167

361

405

410

648


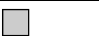

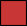

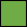

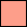


**Phylotype**

C A F D


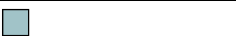

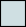

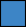

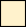

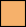

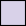

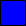

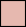

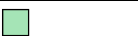

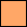

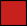


**MIC Values**

0.06-0.125

0.25-4

>4

**CTX-M**

CTX-M-15 CTX-M-27 CTX-M-55

CTX-M-14, CTX-M-15 CTX-M-1

CTX-M-24

CTX-M-3 CTX-M-101


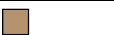

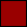


**pAMP-C**

CMY-59 CMY-42


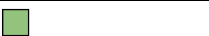

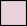

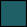

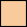

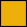

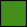

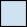

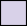

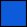

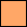


**Carbapenemase** NDM-5 NDM-1 NDM-18

NDM-5 + OXA-244 OXA-484

OXA-48 OXA-181 OXA-244

NDM-1 + OXA-181

NDM-5 + OXA-181


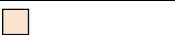

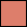


**PBP3 Insert Types**

YRIK YRIN


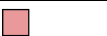

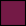

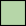

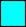

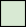

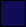

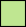

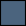

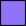

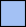

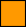


**PBP2**

V229I V522I A543V A530S A388S K572N A543T T173A L378P G601D L573Q

10020

31223

31722

33223

14623

4323

31522

27422

27321

22723

11221

6623

40322

10819

6819

21921

26123

27722

17119

36723

10921

26822

49122

25919

33123

5423

11321

1320

16721

25220

322

15721

12323

19520

25123

9123

33423

19922

17522

14123

5822

22619

30322

20820

16619

15819

31623

420

42622

19223

39523

36223

1220

37822

32322

17023

35223

41022

13723

10919

27719

18219

27819

23919

11019

22419

26521

2421

21922

15521

44922

18621

8921

8721

5219

18619

45722

45622

11222

26723

16522

28622

38223

29922

37323

35323

32221

27622

23022

33821

17619

28823

4319

18523

16819

9922

37422

2622

17020

30622

8422

28719

26819

3623

36822

419

40022

820

320

20122

27219

31922

25722

40122

2019

520

31622

20221

23620

20620

1619

11420

13220

14720

10722

14220
