# Supplementary material for: Decreased susceptibility to cefepime/zidebactam among carbapenemase-producing Escherichia coli from Stockholm, Sweden with alterations in PBP2
Source: J Antimicrob Chemother. 2025 Feb 17;80(4):1137–40. doi: 10.1093/jac/dkaf045 (PMC11962383; doi:10.1093/jac/dkaf045)
Supplement: dkaf045_Supplementary_Data [file dkaf045_supplementary_data.zip › Table_S1_JAC_CLEAN.docx]

Table S1: MICs and key genomic characteristics of the study isolates with V522I substitutions in their PBP2.

| **Strain ID** | **Year of isolation** | **Carbapenemase** | **ESBL** | **PBP3**  **insertions** | **MLST** | **ZID MIC** | **FEP-ZID MIC** |
| --- | --- | --- | --- | --- | --- | --- | --- |
| 17119 | 2019 | NDM-5 | - | YRIN | 167 | 2 | 2 |
| 12323 | 2023 | NDM-5 | CTX-M-15 | YRIN | 648 | 4 | 4 |
| 28622 | 2022 | NDM-5 | CTX-M-15 | YRIK | 405 | 64 | 32 |
| 321 | 2021 | NDM-5 | CTX-M-15 | YRIK | 410 | >64 | >32 |
| 420 | 2020 | NDM-5 | - | YRIK | 648 | >64 | >32 |
| 25220 | 2020 | NDM-5 | CTX-M-15 | YRIN | 167 | 2 | 2 |
| 26723 | 2023 | OXA-244 | - | YRIK | 405 | 0.25 | 0.5 |

ZID, zidebactam; FEP-ZID, cefepime/zidebactam; PBP, penicillin binding proteins.
